# Recent Advances in Nanomaterial-Based Aptasensors in Medical Diagnosis and Therapy

**DOI:** 10.3390/nano11040932

**Published:** 2021-04-06

**Authors:** Olubunmi O. Ayodele, Adeyinka O. Adesina, Sajedeh Pourianejad, Jared Averitt, Tetyana Ignatova

**Affiliations:** Nanoscience Department, The Joint School of Nanoscience & Nanoengineering, University of North Carolina, Greensboro, NC 27401, USA; ooayodel@uncg.edu (O.O.A.); aoadesin@uncg.edu (A.O.A.); s_pouria@uncg.edu (S.P.); jkaveritt@uncg.edu (J.A.)

**Keywords:** aptamer, gold nanoparticles, quantum dots, graphene, MoS_2_, carbon nanotubes, SELEX, biomolecules, diagnosis, signal type, limit of detection

## Abstract

Rapid and accurate diagnosis of various biomarkers associated with medical conditions including early detection of viruses and bacteria with highly sensitive biosensors is currently a research priority. Aptamer is a chemically derived recognition molecule capable of detecting and binding small molecules with high specificity and its fast preparation time, cost effectiveness, ease of modification, stability at high temperature and pH are some of the advantages it has over traditional detection methods such as High Performance Liquid Chromatography (HPLC), Enzyme-linked Immunosorbent Assay (ELISA), Polymerase Chain Reaction (PCR). Higher sensitivity and selectivity can further be achieved via coupling of aptamers with nanomaterials and these conjugates called “aptasensors” are receiving greater attention in early diagnosis and therapy. This review will highlight the selection protocol of aptamers based on Traditional Systematic Evolution of Ligands by EXponential enrichment (SELEX) and the various types of modified SELEX. We further identify both the advantages and drawbacks associated with the modified version of SELEX. Furthermore, we describe the current advances in aptasensor development and the quality of signal types, which are dependent on surface area and other specific properties of the selected nanomaterials, are also reviewed.

## 1. Introduction

Rapid detection of small molecules is of great importance in medical and diagnostic fields, as this allows for early diagnosis of medical conditions. Traditional detection methods such as high-performance liquid chromatography (HPLC) coupled with mass spectrometry is time consuming and requires expensive equipment setup and preparation [1]. The multiplex immunoassay or capillary electrophoresis (CE) technique has the advantage of being fast and highly sensitive but there are setbacks regarding that described in [1]. One of the multiplex immunoassay techniques, enzyme-linked immunosorbent assay (ELISA) is commonly used to detect two or more classes of chemical (antibodies and antigens) simultaneously [2]. However, low antigenicity of small molecules has limited the application of ELISA because antibodies are less sensitive to small molecules [1,3,4]. Other types of biosensors such as surface plasmon resonance biosensor [5,6], polymerase chain reaction (PCR) [7], electrochemical sensors [8] are time consuming because of complex sample and blood culture preparations [9].

The search for alternative recognition molecules has seen aptamers gain prominence due to their ability to detect and bind small molecules [3,10,11,12]. Aptamers, a suitable alternative to antibodies, are chemically derived single stranded DNA (ssDNA) or RNA (ssRNA) with a high capability of folding into secondary or tertiary structures making them recognition molecules with high affinity and specificity to small molecules [1,11]. The possibility of generating aptamers in vitro, which have been pre-matched against target molecules using a synthetic library, have made them useful in early medical diagnosis. In addition, they can be cost effectively synthesized in large quantities and high purity via amplification of selected aptamers by polymerase chain reaction [11,13]. Moreover, the ease of chemical modification of aptamers including their stability at high temperature and pH can be utilized for optimized performance of various biosensor platforms (e.g., flow cytometry, electrochemical sensors, fluorescence microscopy, surface plasmon resonance sensor or lateral flow assays) [14,15]. All these advantages have made aptamers a more robust biosensor than antibodies.

Research on aptamers has been on the increase, resulting in a steady rise in number of publications from 2010–2021. The potential of aptamers as an alternative to antibodies in medical diagnosis has been established by various studies [15,16] and with ease of modification and stability, aptamers can be immobilized non-covalently to nanomaterials to produce biosensors with high specificity and selectivity. As a standalone, nanomaterials can be used as diagnostic devices due to their tunable physical, electrical and chemical properties but their inability to detect small molecules and their non-selectivity towards target biomolecule(s) has limited their adoption in medical diagnosis [17]. Conjugating aptamers with nanomaterials to produce high selective/sensitive biosensors (aptasensors) is now of great interest and importance in medical diagnostics and therapeutics due to their unique properties such as biocompatibility, tunable selectivity, low immune response [17]. Therefore, this review will focus mainly on improvement in aptamer selection via Systematic Evolution of Ligands by EXponential enrichment (SELEX) and the recent advances in fabricating aptamer–nanomaterial hybrids, and their applications as biosensors in Point-Of-Care (POC) diagnostics.

## 2. Selection Protocol

**Figure 1:**
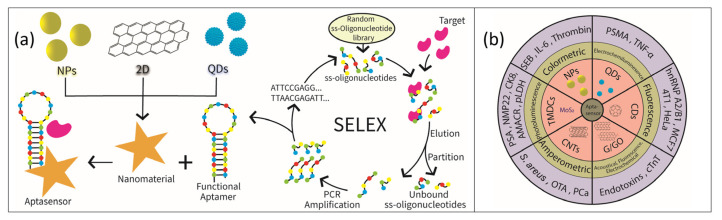
(**a**) In vitro selection of aptamers using the evolutionary method based on Systematic Evolution of Ligands by EXponential enrichment (SELEX). (**b**) Aptasensors designed with nanomaterials with the appropriate signal types.

Aptamers are randomly selected from the database of 1013-1016 single-stranded DNA or RNA oligonucleotides using SELEX [11,18,19]. Figure 1a depicts the concept of in vitro selection of aptamers based on SELEX. Oligonucleotides in the SELEX library typically consist of 40~100 nucleotides, which harbor a random region in the middle and fixed sequences on both ends. Subsequently, the target-binding oligonucleotides are separated from the unbound ones. The bound DNA oligomers are then eluted and amplified by PCR. After several rounds of selection, the resulting DNA sequences (aptamers) with high affinity and specificity are enriched in the pool and sequenced. However, the selection protocol usually requires about 10–15 cycles which could take weeks to actualize the selection process [11,20]. In a bid to circumvent the duration of obtaining aptamers using SELEX, researchers have developed several modified versions of SELEX with mixed success.

Acoustophoresis technique was coupled with the traditional SELEX to obtain a prostate-specific antigen (7 PSA) binding aptamer [21]. This group applied next-generation sequencing (NGS) which helped in accelerating the identification of the screened ssDNA pool, and after the eight cycle of the acoustophoretic SELEX, a 7 PSA binding ssDNA aptamer was obtained and characterized with surface plasmon resonance (SPR) for affinity and specificity. The optimized PSA binding aptamer showed specific binding to PSA with a dissociation constant *K_d_* of 0.7 nM. Recently, acoustophoretic-modified aptamer microbeads were employed for rapid separation and purification of Gram-negative bacteria (GN-B) with some additional washing steps [22] The microbeads with a pore size of 10 µm coated with aptamer had a high affinity for GN-B, and separation performance with high recovery rate (~98%), high purity (~99%) and high volume rate (500 µL/min) made this method an excellent choice in early diagnosis of bacterial infection [22].

Capillary electrophoresis–systematic evolution of ligands by exponential enrichment (CE–SELEX) is primarily an electrophoretic mobility technique used for ion separation, thus generating aptamers with a high specificity property and short selection rounds [23]. Yang and Bowser [24] applied the CE-SELEX method to select a catalytic DNA aptamer which resulted in high-nanomolar to low-micromolar dissociation constants after only three rounds of selection. The aptamer was selective towards a small-molecule target, N-methyl mesoporphyrin (NMM), with a molecular weight of only 580 g/mol. An improved CE-SELEX, fractional collection approach in CE-SELEX (FCE-SELEX) was developed by Zhang’s group [25]. Their approach integrated fraction collection with a facile oil seal method thus eliminating contamination, and this resulted in a single round selection of amplified DNA–target complex, a streptavidin-binding aptamer (SBA).

Non-suitability and affinity of CE-SELEX for small molecules led to the development of Cell-SELEX which utilizes live cells during the aptamer selection protocol. In a recent study, a whole living cell was utilized as a target in Cell-SELEX selection, yielding aptamers selected from membrane proteins in their indigenous configuration. More so, the technique provides a protocol to purify and identify diagnostic cell-surface biomarkers [26]. However, this process is limited by high selection rounds (~15 rounds) and the occurrence of non-specific interactions. In a bid to reduce the selection round and non-specificity, Ray and White [27] devised a method where an additional selection pressure was applied with RNAse to isolate surface-binding aptamers thus aiding selection of cell-internalizing aptamers. After seven rounds of Cell-SELEX against human pancreatic cancer cell lines (MiaPaCa-2), the selected pool of RNAs sequence were not specific for MiaPaCa-2 due to the formation of a structural motif that binds strongly to the selected aptamer sequence. The authors proposed removal of the structural motif sequence during Cell-SELEX in order to improve specific binding of aptamers to their target molecules (cells).

Several other modified SELEX developed since 2015 to date are also presented in Table 1.

## 3. Nanomaterials Based Aptamer Sensors as Diagnostic Tool

Aptamers are chemically derived single strands of either DNA or RNA oligonucleotides that can be conjugated with various types of nanomaterials to produce POC aptasensors capable of detecting small molecules or biomarkers. In the conjugated device, the aptamer serves as a highly sensitive and selective recognition element while nanomaterials present high surface area and excellent optical, electrical and electrochemical properties rendering them as suitable and highly sensitive transducers [32,33]. The signals generated via the binding of small molecules by the aptasensors can be optical, colorimetric, electrochemical, fluorescence, surface-enhanced Raman spectroscopy/scattering (SERS), surface plasmon resonance (SPR) signals [34,35] and these types of signals are sometimes dependent on the nature and properties of the adjunct nanomaterials. Figure 1b presents selected nanomaterials as transducers which are dependent on detection signal type. Current research on application of nanomaterials as transducers in aptasensors and their dependency on types of detection signal will be the focus of the next chapter and a summary is provided in Table 2.

### 3.1. Aptamer–Gold Nanoparticle Aptasensors

**Figure 2:**
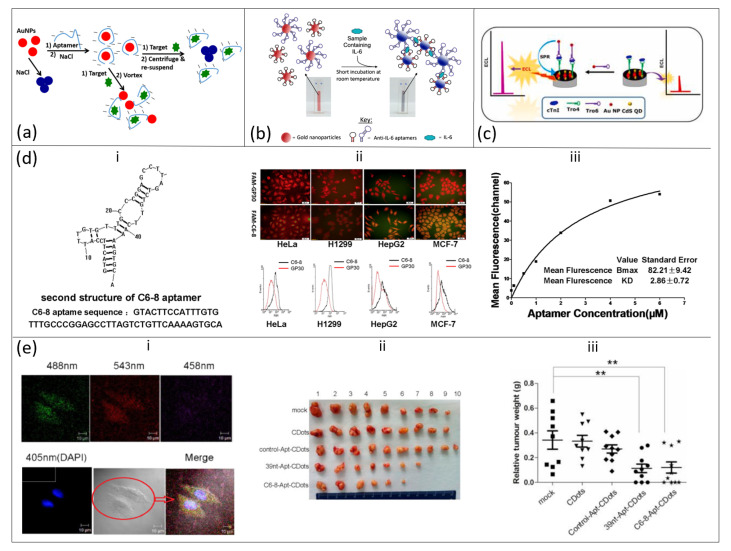
Application of aptamer-gold nanoparticles (AuNPs)/quantum dots (QDs)/carbon nanodots(CDs) conjugates in medical diagnosis. (**a**) Schematic representation of AuNPs function in a colorimetric aptasensor. This image was adapted from Alsager et al. [36] with permission from *Nature*. (**b**) Detection of interleukin-6 using a colorimetric aptasensor. Aptamers with two complimentary IL-6 target moieties were coated on AuNPs and exposed to protein mixtures containing IL-6. Aggregation of AuNPs, vis-a-vis color changes, occurred via Van der Waal binding of IL-6 with the aptamers sandwiched with the two complimentary IL-6 moieties. This image was adapted from Giorgi-Coll et al. [37] with permission from Springer Nature. (**c**) Schematic representation of an electrochemiluminescence (ECL) aptasensor based on conjugation of CdS QDs and cardiac troponin aptamer (Tro6). This image was adapted from Kitte et al. [40] with permission from Elsevier. (**d**-**i**) The sequence and secondary structure of C6-8 aptamer isolated using the Cell SELEX procedure. (**d**-**ii**) Selection of C6-8 aptamer based on its affinity for multiple tumor cell lines as revealed by fluorescence micrographs. Below: flow cytometry analysis revealing specific binding preference of C6-8 aptamer to HepG2, MCF-7, H1299, and HeLa tumor cells. Inset: (**d**-**iii**) The binding affinity, as measured by fluorescence microscopy, of aptamer to representative HeLa cell. (**e**-**i**) Confocal micrograph of CDs–C6-8 aptamer targeting and binding tumor cells inside the nuclei. (**e**-**ii**,**e**-**iii**) images and graph showing the CDs–C6-8 aptamer’s ability to inhibit proliferation of lung cancer cells, hepatocarcinoma (GFP-HepG2), at each point time. Reprinted with permission from Elsevier [42].

A family of Gold Nanoparticles (AuNPs) can exist in multiple dimensions and, based on shape, they can represent (i) one-dimensional (nanorods, nanowires, nanotubes, etc.); (ii) two-dimensional (squares/rectangles, pentagons, stars, etc.) and, (iii) three-dimensional (nanocubes, nanopiramids, nanospheres) materials. AuNPs have unique physical and chemical properties, with their localized surface plasmon resonance (LSPR) property providing colloids extinction coefficients (EC) greater than the EC of conventional dyes [49]. A distinct color in the visible spectrum could be observed when there is a change in the dispersion–aggregation state of AuNPs [49,50], and the resultant nanoclusters of different sizes respond differently to the wavelength of light scattering [51,52,53]. In addition, the color change could also be due to a change in surface charge of AuNPs to neutral. Thus, in the presence of a target analyte, a chemical interaction can occur with the particles (i.e., AuNPs) leading to a change in color usually from red to blue [36,54] A schematic illustration of dispersion and aggregation of functionalized gold nanoparticles after exposure to a target (small molecule) is shown in Figure 2a [36]. Taking advantage of this unique property of AuNPs, aptasensors consisting of aptamer–AuNP conjugates can be constructed for rapid detection of molecules of medical significance.

A colorimetric assay of Staphylococcal Enterotoxin B (SEB) was adopted for aptamer–AuNP conjugates and changes in color were observed by naked eyes or spectrometrically with a linear response in the range of 50 µg/mL–0.5 ng/mL and a limit of detection (LOD) of 50 ng/mL [36]. Based on the discovery of colorimetric assay of aptamer–AuNP conjugates in detection of small molecules, Giorgi-Coll et al. [37] used the colorimetric concept of aptasensors to detect the immuno-signaling molecule interleukin-6 (IL-6), a diagnostic marker of meningitis (Figure 2b). Two complimentary aptamers with each different IL-6 target moiety were conjugated with AuNPs and upon introduction into mixed protein solution with IL-6 molecules, binding of IL-6 to the complimentary strands occurs leaving AuNPs to aggregate. The aggregation of nanoparticles caused a visible color change from red to blue and a change in absorption maximum from 520 to 540 nm was also observed. They further reported the sensitivity of the aptasensor in the range of 3.3–125 µg/mL with an LOD of 1.95 µg/mL. Peng et al. [38] also developed a colorimetric aptasensor capable of detecting thrombin, an endoprotease responsible for blood clotting. When the thiol-modified 27-mer DNA oligonucleotides (anti-thrombin aptamer) were conjugated with AuNPs, detection of thrombin molecule occurred, albeit at a higher concentration, via aggregation of aptamer–AuNP complexes. Binding of thrombin molecules to aptamer–AuNP complexes led to visible color change from red to blue, even at a low concentration of 5 pM (1.679 ng/mL) and the intensity is predicated by distance-dependent optical properties of AuNPs.

The concentration dependence of colorimetric aptasensors (Apt–AuNP conjugates) has limited their utilization in early detection of biomarkers. For example, Peng and co-workers were only able to achieve detection of thrombin at a high concentration of 5 pM [38]. This limitation encouraged Chen et al. [39] to engineer a “sandwich-type” electrochemical-based aptasensor. The sensor was developed by incorporating two thrombin aptamers (TBA1 and TBA2) onto AuNPs that were previously incubated with [Ru(NH_3_)_6_]^3+^. The high aptamer loading capacity of TBA1 and TBA2 onto AuNPs helped to improve signal amplification while [Ru(NH_3_)_6_]^3+^ aided signal conversion. The engineered electrochemical aptasensor was able to detect thrombin at a linear range of 1 fM to 6 pM and limit of detection was found at 0.1429 fM (S/N = 3) under optimized conditions.

Some of the drawbacks of colorimetric signal method could be resolved with either surface plasmon resonance (SPR) or Surface-enhanced Raman spectroscopy (SERS) due to their ability to amplify optical signals within the SERS hotspot between AuNPs and the Au surface [55]. Zhang et al. [56] utilized SERS signal type to construct an aptasensor based on AuNPs modified with Raman molecules (Mercaptobenzoic acid and 5,5′-Dithiobis(2-nitrobenzoic acid) and it was applied for the detection of *Salmonella typhimurium* and *Staphylococcus aureus*. The Raman enhanced spectra were quantified to obtain linear range of detection between 10^2^ and 10^7^ cfu/mL and LOD of 15 cfu/mL for *S. typhimurium* and 35 cfu/mL for *S. aureus*. Despite the sensitive nature of aptamer–AuNPs biosensors, incomplete dissociation of excess non-target binding nucleotides resulting in non-aggregation of particles has been a major drawback of the AuNPs-based aptasensor.

### 3.2. Quantum Dot-Based Aptasensor

Quantum dots (QDs) are new classes of zero dimensional (0D) semiconductor nanomaterials (mainly Cadmium telluride [CdTe], Cadmium selenide [CdSe] and CdTeSe alloy) with exceptional optical properties [57], high quantum yield [17], high resistance to degradation and photobleaching [17,58] and narrow fluorescence emission and photoluminescence spectra [58,59]. In addition, QDs are excellent fluorescence resonance electron transfer (FRET) donors–acceptors; this property is observed in QDs modified with aptamers. This ability is useful for sensors in medical POC diagnosis [17]. In 2007, Jon and coworkers synthesized a novel quantum dot (QD)−aptamer (Apt)−doxorubicin (Dox) conjugate (QD−Apt(Dox)) and was used as a detection platform for cancer sensing and therapy [59]. The surface of QDs was immobilized with A10 RNA aptamer which was followed by intercalation of Dox onto the other double-strand of A10 aptamer thus creating a donor–acceptor FRET platform between QDs and Dox. This system can recognize and imaging prostate cancer cells that express prostate specific membrane antigen (PSMA) protein through delivery of Dox via activation of fluorescence QD. Recently, FRET aptasensors synthesized by conjugating Apt–QDs–AuNPs have been used for the detection of Staphylococcus aureus [60] and Tumor Necrosis Factor-alpha (TNF-α) [61]. The authors observed that selective binding occurred with limit of detection of 2 cfu/mL for S. aureus and strong fluorescence of an aptamer–QD conjugated donor was successfully quenched by an AuNPs acceptor in the presence of target molecules.

Target-specific Apt–QDs sensor probes have also been extended to electrochemiluminescence (ECL), chemiluminescence, electrochemical and photoelectrochemical detection methods [62]. For instance, Jin’s group developed a highly sensitive ECL aptasensor by conjugating CdS QDs (as an ECL luminophores source) and AuNPs (as an ECL plasmon source) with Tro4 and Tro6 sandwiched type aptamer (Figure 2c) [40]. The aptasensor was capable of detecting cardiac troponin 1, a biomarker for acute myocardial infarction, with an LOD of 0.75 fg/mL and they further observed that the signal of the surface plasmon enhanced electrochemiluminescence (SPEECL) aptasensor was five-fold higher with AuNPs as the ECL plasmon source. Isildak et al. [41] also investigated detection of thrombin by an ECL aptasensor. When CdS nanocrystals (CdS NCs) and luminol were conjugated with aptamer/AuNPs, detection of thrombin was based on ratiometric ECL and increase in thrombin concentration led to a decrease in intensity (quenching) of CdS NCs with concomitant increase in luminol intensity. An LOD of 500 fg/mL was achieved with the ratiometric method. Selective and sensitive detection of molecules by an ECL aptasensor is highly anticipated to be a device of choice in PoC diagnostic tools; however, high cost and inaccessibility of ECL equipment have been major limitations in its adoption.

### 3.3. Carbon Quantum Dot-Based Aptasensor

Carbon quantum dots (CQD), also known as carbon nanodots, are a second example of 0D nanomaterials with sizes less than 10 nm and possess excellent properties such as efficient fluorescence emission, low toxicity, high solubility in water, high quantum yield, high photo stability, broad excitation spectrum [63,64,65], they are great candidates in sensor/biosensor applications. The optical absorption of CQDs is primarily due to their electronic transition from π to π* caused by the presence of phenyl groups and C=C bonds or from n to π* of C=O bonds, and thus exhibiting absorption in the near-ultraviolet region and weaker absorption intensity at the visible to near-infrared (NIR) regions [66,67]. CQD-based sensors are highly sensitive with LOD in the range of nanomolar to picomolar but occasionally in the femtomolar range; their mechanism of action could be based on energy transfer, FRET, fluorescence/static/dynamic quenching or photo-induced electron transfer [68]. As a standalone, fluorescent CQDs have been used to detect metal ions, amino acids and adrenaline with LOD at 0.05–90 nM [69], 30 µM [70] and 10–100 µM [71], respectively. In the last decade, sensitivity and selectivity of biosensors derived from CQDs have been improved with the introduction and conjugation of aptamers with CQDs [72,73].

The diagnostic and therapeutic uses of carbon nanodots (CDs)–aptamer conjugates in multiple tumor cells were initially established using SELEX to select an aptamer targeting ROS17/2.8 (rat osteosarcoma) cell [74] and then followed by conjugation of CDs to C6-8 aptamer thereby conferring the CDs-conjugated aptamer the ability to freely enter multiple living tumor cell lines (HepG2, MCF-7, H1299, and HeLa) (Figure 2d,e) [42]. The binding of synthesized C6-8 aptamer to the aforementioned cancer cell lines was confirmed by fluorescence-microscopy and flow cytometry and a specific binding to target hnRNP A2/B1, a major component of the heterogeneous nuclear ribonucleoprotein core complex, was observed. The therapeutic effect of conjugated fluorescent CDs and the C6-8 aptamer was based on the ability of the conjugate to target the tumor cell (hnRNP A2/B1protein) which is located in the nucleus. Meanwhile, 39 nt–Apt–CDs, regarded as a positive control to the CDs–C6-8–Apt, showed high affinity for cytoplasm of tumor cells; however, control groups (control Apt–CDs and CDs) exhibited either weak or no binding to the tumor cells. The CDs–C6-8 aptamer was able to inhibit growth of the tumor both in vitro and in vivo, and subsequently produce a reduction in the relative weight of tumor cells.

Further to the detection of cancer, Motaghi et al. [75] developed an aptasensor based on the nanoconjugation of carbon nanodots (CDs) and a nucleolin aptamer, AS1411. The spectrofluorometric aptasensor, used as a probe for the detection of mouse breast (4T1), human breast (MCF7), and human cervical (HeLa) cancer cells, was sensitive to detect cancer cells through overexpression of AS1411 on the cells’ surface, thereby causing the release of CDs which are measured by the intensity of fluorescence. The process is relatively simple, inexpensive and offers high sensitivity with a detection threshold of ~100 cells/mL. Based on this process, Kong and co-workers’ modification of a CDs–AS1411 aptamer with polyethyleneimine (PEI), (CDs–PEI–AS1411) led to a slightly improved sensitivity for detection of MCF-7 cancer cells [76].

### 3.4. MoS_2_-Based Aptasensor

Two-dimensional molybdenum disulfide (2D MoS_2_) belongs to a 2D transition metal dichalcogenides family. 2D MoS_2_ exhibits many fascinating properties: optical, electrochemical, catalytic, electronic, which are highly dependent on the number of atomic layers. It is a semiconductor nanomaterial with direct band gap of *1.8 eV,* which makes it suitable for optoelectronic nanodevices [77]. Likewise, its excellent photoluminescence, FRET, large surface area and carrier mobility properties have made it a good candidate for sensor applications [77,78]. However, to improve the biosensing capability of MoS_2_, the basal surface can be functionalized to respond specifically and selectively to a target molecule [79]. Biofunctionalization of MoS_2_ improved detection of various cancer biomarker proteins, e.g., prostate-specific antigens [80], nuclear matrix protein 22 (NMP22) and cytokeratin 8 (CK8) [81] or enzymes, e.g., Alpha-methylacyl-CoA racemase (AMACR) [82] and, as such, an improved low LOD was reported. Despite an improvement in sensitivity of MoS_2_-based biosensors, selective detection of a particular biomarker is still a difficult task to resolve, thereby requiring a blocking agent to prevent non-specific binding during sensing reactions [83].

**Figure 3:**
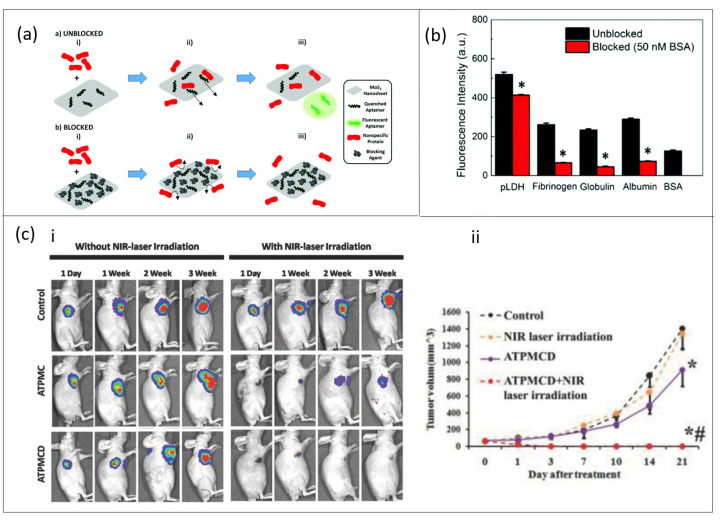
Strategy of enhancing sensing capabilities of MoS_2_-based aptasensors in medical diagnosis. (**a**,**b**) The effect of surface blocking on non-specific fluorescence recovery in a fabricated MoS_2_-based fluorescence resonance electron transfer (FRET) aptasensor and its corresponding effect on fluorescence intensity observed after the addition of protein to either an aptasensor in unblocked or blocked format. Adapted from Geldert et al. [86] with permission from Royal Society of Chemistry. (**c**) Therapeutic efficacy of ATPMC and ATPMCD in inhibiting cancer tumor growth. (**c**-**i**) Monitoring of tumor tissues following injection with physiological saline via signal intensity of noninvasive bioluminescent imaging (BLI). (**c**-**ii**) Tumor volumes calculated based on sizes of tumor tissues in mice after exposure to different treatment methods (* *p* < 0.01 compared with control; # *p* < 0.01 compared with ATPMCD group). Adapted from Meng et al. [87] with permission from Wiley GmbH.

The selectivity of a MoS_2_-based biosensor is readily improved by conjugation with ssDNA aptamers due to spontaneous adsorption via van der Waal interaction between nucleobases of ssDNA and the basal plane of 2D MoS_2_ [84,85]. Following this assumption, Kong et al. [43] synthesized a novel aptamer-functionalized MoS_2_ fluorescent sensor and, when applied to detect PSA in human serum, a decent selectivity was obtained with a detection limit of 0.2 ng/mL. Thereafter, different approaches to improve selectivity and sensitivity of MoS_2_-based aptasensors such as the surface blocking strategy [86] and dual signal amplification strategy [44] have been developed. By design, sensing specificity of a MoS_2_-based FRET aptasensor towards malarial biomarker Plasmodium lactate dehydrogenase (pLDH) was enhanced by a surface blocking strategy [78]. The surface blocking mechanism, implemented using Bovine Serum Albumin (BSA), ensured an increase in specific pLDH recovery which led to an improved signal-to-noise ratio as compared to the unblocked samples (Figure 3a,b).

Most studies have focused on the diagnostic function of MoS_2_-based aptasensors [88,89], but the therapeutic effect of these aptasensors is also very important since MoS_2_ has been labeled as being of low toxicity relative to other kinds of nanomaterials [79]. In a recent study, Dong and co-workers investigated both the diagnostic and therapeutic capability of an MoS_2_-based aptasensor [87]. A pre-conjugated polyethylene glycol (PEG) sample and MoS_2_ decorated with Cu1.8S nanoparticles was functionalized with aptamer (AS1411). The nano-aptasensor (ATPMC) was capable of photoluminescence, photoacoustic and photothermal imaging of tumor cells and it also enabled selective gene probe delivery which aided in detection of intracellular microRNA expressed in cancer cells and doxorubicin (DOX) for chemotherapy. Furthermore, the antitumor efficiency of ATPMC could be enhanced by loading the aptasensor with DOX (ATPMCD) and thereafter triggered with NIR targeted chemo–photothermal combined therapy (Figure 3c). It can be emphasized that MoS_2_-based aptasensors will provide the needed sensitivity and selectivity required in biosensors and their potential in therapy applications, however difficulty in synthesis of continuous and defect-free MoS_2_ is a major drawback and further research is needed in this area.

### 3.5. Carbon Nanotube-Based Aptasensor

The carbon nanotube (CNT) is the most famous member of the one-dimensional class of nanomaterials. CNT can be visualized as a graphite layer rolled into cylinder of a few nanometers diameter. Compared to other nanomaterials, CNTs possess a unique combination of optical (near infrared luminescence), electrical (high mobility, high conductivity, higher electron transfer kinetics) and chemical (extremely high surface area 1300 m^2^/g, ability to be functionalized) properties which make CNTs suitable for various biomedical applications [90,91], A recent study investigated the binding specificity of Staphylococcus aureus by CNTs hybridized with 1-pyrenebutanoic acid succinimidyl ester (PBASE) where a moderate LOD of 4 log CFU/mL was achieved [92]. Immobilization of biomolecules on the surface of CNTs sometimes requires chemical modification, which may interfere with the electrical properties of CNTs [92,93].

**Figure 4:**
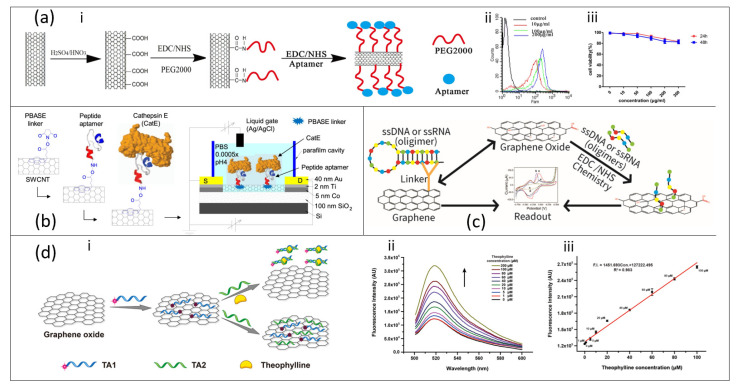
Aptasensors constructed by conjugation of carbon nanotubes (CNTs) with aptamers. (**a**-**i**) Stepwise synthesis of aptasensor based on conjugates of CNTs, polyethylene glycol (PEG) and aptamer, (**a**-**ii**) the cellular uptake of a CNT-based aptasensor at different concentrations monitored by flow cytometry, (**a**-**iii**) concentration dependency of the aptasensor toxicity in cells. Adapted from Gu et al. [96] with permission from Springer. (**b**) Experimental setup for the immobilization of peptide aptamer onto the surface of a CNT and the operating set-up of the liquid-dated CNT FET device for CatE detection. Adapted from Tung et al. [45] with permission from Springer Nature. (**c**) Schematics of the mechanism of aptasensing of biomolecules on graphene surfaces. (**d**-**i**) Schematic illustration of the sensing mechanism of a self-assembling Graphene oxide/RNA-based aptasensor for the turn-on detection of theophylline in serum, (**d**-**ii**,**d**-**iii**) the fluorescence spectra of a GO/RNA-based aptasensor in response to different concentrations of theophylline in serum and its subsequent linear relationship between fluorescence intensity and theophylline concentration (1–100 μM). Adapted from Ling et al. [47] with permission from Elsevier.

The debilitating effect of chemical modifier can be alleviated by introducing aptamers onto the CNT–PBASE matrix, thereby achieving an aptasensor with high specificity and selectivity, and coupled with high linear range of detection and low LOD. For instance, Tung and co-workers fabricated a liquid-gated CNT FET aptasensor modified with Cathepsin E-binding peptide aptamers as a POC platform for the detection of serum Cathepsin E (CatE) disease biomarkers in breast cancer patients (Figure 4b) [45]. The CNT was functionalized by a peptide aptamer through PBASE linker. The aptasensor was found to be highly selective, and label-free detection of CatE at low concentrations in both phosphate-buffered saline (2.3 pM) and human serum (0.23 nM) was obtained. In addition to the photoluminescent properties of CNTs that assist in their near infrared monitoring, their electrochemical properties can also be employed based on amperometric, potentiometric and conductometric nature [94]. An amperometric aptasensor based on conjugation of CNTs, complementary DNA and methylene blue resulted in rapid detection of mycotoxin ochrotoxin A (OTA) with high specificity and low LOD within 52–134 pM in serum and juice spiked with OTA [95].

Over the years, researchers have explored CNT in gene delivery and other applications related to diagnosis and therapy [97,98,99]. Complementing CNTs with aptamers would not only produce superior biosensors but also effective therapeutic tools in medical parlance. As a brief study, Gu and co-workers studied CNTs conjugated with anti-PSMA aptamer and PEG as a nanoultrasound contrast agent (Figure 4b) and they found out that the contrast has superior visuality, good distribution and development effect. The conjugated CNT/aptamer was also capable of specific targeting of prostate cancer (PCa) cells due to good cell uptake [96]. However, CNTs, due to their ability to pass through the blood–brain barrier, are toxic to organs and can induce death at very high dosage but, at low dosage, they can serve as therapeutic drug delivery agents [99]. The toxicity of CNTs can be caged with PEG resulting in a noticeable decline in toxicity even at higher concentration and prolonged time (Figure 4a).

### 3.6. Graphene/Graphene Oxide-Based Aptasensor

Recently, atomically thick graphene-based materials have received extensive research attention in the biosensor field. The sp^2^-hybridized carbon atoms in the two-dimensional structure of graphene can easily be functionalized. In addition to specificity, graphene has excellent electrical, electrochemical, physicochemical properties and its high sensitivity to external stimulus which make it an exceptional candidate for optoelectronics sensors [100]. Graphene and its derivatives, in either their non-functionalized or functionalized state, can be used to selectively and sensitively detect biomolecules [101,102,103,104,105]. However, performance of biosensors fabricated from graphene/GO can be further improved with the introduction of molecule-specific aptamers.

Due to the unique structural configurations of graphene, ssDNA aptamers can either bind to graphene surface via electrostatic interaction with DNA bases or via non-covalent π–π interaction [1,106]. Thus graphene-based aptamer sensors give rise to a high density platform for biomolecule immobilization allowing detection of a wide range of targets (Figure 4c). The detection of biomarkers could be in vitro as well as in vivo since graphene can act as a delivery vehicle for aptamers into living cells or animals [1]. An in vitro detection of endotoxin, a complex lipopolysaccharides found in cell walls of Gram-negative bacteria, was performed using a highly sensitive and label-free shear horizontal surface acoustic wave (SH-SAW)/single-layer graphene (SLG)-based aptasensor [46]. The SH-SAW/SLG aptasensor demonstrated a linear relationship with the endotoxin concentration range from 0 to 100 ng/mL and reached a detection limit of 3.53 ng/mL. Moreover, the stability and excellent specificity make the SH-SAW biosensor a promising alternative to conventional methods for detecting endotoxins.

The fluorescence quenching property of graphene and GO make it a strong acceptor for fluorescence resonance energy transfer (FRET) owing to its broad absorption in full visible spectrum [107]. For instance, the quenching ability of GO was utilized to detect theophylline in serum with a self-assembling RNA aptamer (33-amer) conjugated on GO by π–π stacking interaction. The aptasensor resulted in larger range of detection (1–100 µM to 0.1–10 µM) primarily due to lack of interference with fluorescence intensity by GO or other environmental factors [47] (Figure 4d). A electrical aptasensor synthesized by dielectrophoretic deposition of GO on a polyethylene terephthalate (PET) substrate and its subsequent reduction to reduced-graphene oxide (rGO) provided an efficient platform for the detection of cardiac biomarker, Cardiac troponin T (cTnT), with a linear range 0.001–10 ng/mL and LOD between 1.2 and 1.7 pg/mL [48]. Other aptasensors based on either a electrochemical graphene field effect transistor (GFET) [10,108] or voltametry [109] have shown good specificity signal response to an analyte of interest. Graphene and its derivatives have the potential to revolutionize the biosensor industry because they are readily scalable and cost effective based on the chemical vapor deposition (CVD) production technique; however, transferring pristine graphene devoid of contaminants and defects has been a major challenge in the R&D sector.

### 3.7. Other Nanomaterial-Based Aptasensor

In addition to the previously reviewed nanomaterial-based aptasensors, it is pertinent to discuss briefly other nanomaterials with properties suitable for biosensing applications. Some of these materials were conjugated directly with the aptamer [110] or functionalized with other types of nanomaterial before conjugation with aptamer [111,112]. For instance, dispersed silver nanoparticles (AgNPs) were conjugated with an aptamer for colorimetric detection of adenosine in urine samples of cancer patients. A linear range of detection of 60–280 nM with 21 nM LOD was reported and the repeatability of the aptasensor was confirmed with urine samples of cancer patients with percent recoveries within 98–107% [113]. In the second instance, Li et al. [114] achieved detection of adenosine triphosphate (ATP) using an aptasensor derived from tungsten disulfide (WS*_2_*) immobilized on the -SH end of an ssDNA sequence which was previously conjugated with an Au electrode (SH-DNA/Au/WS_2_).The detection of ATP was possible over a concentration range of 0.1 µM to 5 mM, and an LOD of 1.5 nM. Several types of ultrasensitive aptasensors have also been fabricated for medical diagnosis (Table 3). In addition, some of these nanomaterials outperform commonly used materials such as AuNPs, MoS_2_ and graphene due to their unique properties. For example, WSe_2_ possesses superior electrical conductivity over MoS_2_ due to the metallic nature of Se and therefore it is more suitable in electrochemical sensing applications [112].

## 4. Future Perspectives and Conclusions

The gradual rise in the application of biosensors in small molecule detection has made diagnosis and therapy of early onset of medical conditions an exciting possibility. Aptamer, an artificial single stranded DNA (ssDNA) or RNA (ssRNA) was synthesized due to limitations in antibodies’ sensitivity to small molecules and complex setup of chromatographic detection methods. Since aptamers can be chemically synthesized and modified to detect specific biomarkers, they are now becoming the preferred tool for diagnosis of medical conditions. The selection protocol of these aptamers is based on SELEX which enables the isolation, purification, and amplification of target-binding oligonucleotides. However, the protocol could sometimes be actualized after 10–15 selection cycles and some improved methods were proposed with mixed results. The short half-lives of aptamers are a major challenge in biosensor industries. An improved performance in aptamers’ selectivity and sensitivity is achieved upon conjugation with nanomaterials (aptasensor), which are themselves excellent sensing materials with tunable properties. At the same time, high sensitivity of nanomaterials can bring additional challenges; for instance, tendency for self-agglomeration resulting in modulation of optical response. The aptasensors can bind small molecules with very low LOD ranging from nanomolar to femtomolar and the signals generated are dependent on the nature of the nanomaterials. Some research studies also confirmed the therapeutic potentials of aptasensors especially in inhibiting tumor growth but some of the setbacks include toxicity to human cells.

Due to technological advancement in nanomaterial synthesis and control, we foresee fabrication of aptasensors as portable medical devices that will be capable of detecting early biomarkers of disease condition and simultaneously be used as benign therapeutics.

## Figures and Tables

**Table 1:** A short description of selected modified SELEX stating their key advantages and drawbacks.

Modified SELEX	Description of Modification	Selection Rounds	Mean K_d_ (nM)	Advantages	Drawbacks	Reference
Hi-Fidelity SELEX	Hi-Fi SELEX utilized a fixed-region blocking elements to safeguard functional diversity of the SELEX library. The chemistry of aptamers is engineered such that non-specific retention of aptamers is strongly inhibited by modification of the target-display surface and composition of the equilibration solvent. Integration of novel qPCR into the Hi-Fi SELEX workflow allowed for rapid sequencing during selection rounds.	3 selection rounds. 10^7^–10^8^	~2 and 20	Partition efficiencies approaching 10^6^ are realized. High potential value in screening a small amount of retained aptamers for putative therapeutics.	High reagent volume is required to sufficiently amplify library members between each selection round.	[28]
HT-SELEX	High throughput sequencing technology and bioinformatics analysis coupled with SELEX (HT-SELEX) assisted in understanding the effect of initial library and PCR methods in the RNA aptamer identification. The analysis revealed that a distinct sequence and nucleotide existed in the initial, unselected libraries and the fate of “biased sequences” was target-dependent during selection. Amplification by either PCR-driven SELEX or droplet digital PCR (ddPCR)-driven SELEX did result in molecular evolution, during which highly enriched aptamers were produced after the 5th round of selection.	5–7 rounds	PCR-driven SELEX = 65.2 ddPCR-driven SELEX = 111.2	ddPCR-driven selection allowed preservation of molecular diversity and chances of obtaining highly structural sequences are increased.	ddPCR requires extra steps: (1) droplet generation, (2) extraction of the amplicon by organic solvent.	[29]
Click SELEX	A chemical modification of nucleic acid libraries carried out using copper-catalyzed alkyne-azide cycloaddition (CuAAC) or click chemistry allowed for the introduction of a wide range of possible functionalities. The interaction properties of the resultant DNA aptamers are not accessible with the cononical set of nucleotides. The modified DNA is incubated with the target molecule and the best binding sequences are recovered after subsequent selection sequence. The chemical modification is removed during the amplification process.	15 cycles, ~1 day for each selection cycle	_	Relies only on well-established and commercially available building blocks. This feature makes click-SELEX accessible to many laboratories, even if in-house synthesis is not available.	The azide of choice must be stable under the conditions used for CuAAC and during the selection process; in addition, it must quantitatively react with the alkyne-modified DNA strand to avoid non-functionalized nucleobases during the selection process.	[30]
Cell-SELEX	A differential binding Cell-SELEX workflow that adapts the *FASTAptamer* toolbox and bioinformatics *edgeR* is employed to achieve more informative metrics about the selection process. The high-throughput (HT) aptamer identification method is coupled with the Cell-SELEX technique to increase the aptamer selection rate against live cells.	11 selection cycles	_	Shorter time for aptamer identification. Selection of aptamer sequences that can selectively bind to the target and control cells.	High round of selection cycles, at the 11th round, aptamer’ binding was non-specific.	[31]

**Table 2:** Summary of aptasensors based on commonly used nanomaterials.

Aptasensor	Signal Type	Target Molecule	Linear Range	Detection Limit	Reference
AuNPs-SEB aptamer	Colorimetry	Staphylococcal enterotoxin B	50 µg/mL–0.5 ng/mL	50 ng/mL	[36]
AuNPs-IL-6 aptamer	Colorimetry	Interleukin-6	3.3–125 µg/mL	1.95 µg/mL	[37]
AuNPs-thio/27-mer aptamer	Colorimetry	Thrombin	5 pM–2 nM	5 pM	[38]
AuNPs-[Ru(NH_3_)_6_]^3+^-TBA2 aptamer	Electrochemical	Thrombin	1 fM–6 pM	0.1429 fM	[39]
CDS-QDs/AuNPs/Tro6 aptamer	Electrochemiluminescence	Cardiac troponin 1	1 fg/mL–10 ng/mL	0.75 fg/mL	[40]
CdS-NCs/AuNPs/luminol aptamer	Ratiometric ECL	Thrombin	-	500 fg/mL	[41]
CDs/AS1411 aptamer	Spectrofluorometry	Cancer cells	-	~100 cells/mL	[42]
MoS_2_-NS aptamer	Fluorescence	PSA		0.2 ng/mL	[43]
MoS_2_-AuNPs/TiONBs/MC-LR aptamer	Electrochemical	Microcystin-LR	0.005–30 nM	0.002 nM6	[44]
SWCNTs-PBASE aptamer	FET	Capthepsin K	2.3 pM–0.23 nM	-	[45]
Graphene/SH-SAW aptamer	Surface Acoustic Wave	Endotoxins	0–100 ng/mL	3.53 ng/mL	[46]
GO/33-mer aptamer	Fluorescence	Theophylline	1–100 µM	0.155 µM	[47]
rGO-PET/cTnT aptamer	Electrical	Cardiac troponin T	0.001–10 ng/mL	1.2–1.7 pg/mL	[48]

**Table 3:** Aptasensors fabricated on other types of nanomaterials.

Aptasensor	Signal Type	Target Molecule	Linear Range	Detection Limit	Reference
Tungsten diselenide/AuNPs based- thrombin aptamer (WSe_2_/AuNPs/TBA1 apt)	Electrochemical	Thrombin	0–1 ngmL^−1^	190 fgmL^−1^	[112]
Streptavidin-conjugated fluorescent silica nanoparticles-based biotin aptamer (SA-FSiNPs/Bio-TLS11a apt)	Fluorescence	HepG2 cell	-	-	[115]
Amino- and carboxyl-modified silica-coated terbium (III) thiacalix[4]arenesulfonate-based Sgc8 aptamer ([Tb(TCAS)]-SiNPs/Sgc8 apt)	Luminescence	Leukemia cell	-	-	[116]
Molybdenum diselenide modified AuNPs-based ochratoxin A aptamer (MoSe_2_/AuNPs/OTA apt)	Electrochemical	ochratoxin A	0.0001–1 nM	0.08 pM	[117]
Tungsten disulfide nanosheets/Au nanoparticles-modified glassy carbon electrode -based estradiol aptamer (GC-WS_2_/AuNPs/estrad apt)	Electrochemical	17b-estradiol	1.0 × 10^−11^–5.0 × 10^−9^ M	2.0 × 10^−12^	[118]
Vanadium disulfide-based cytochrome c aptamer (VS_2_/Cyt c apt)	Fluorescence	Cytochrome c	0.75 nM–50 µM	0.5 nM	[119]
Cobalt sulfide/Au nanoparticles modified electrode-based 17β-estradiol aptamer (CoS/AuNPs/17β-estrad apt)	Electrochemical	17β-estradiol	1.0 × 10^−9^ −1.0 × 10^−12^ M	7.0 × 10^−13^ M	[111]
Acetylene black-copper sulfide nanosheets/Au modified electrode-based DNA aptamer (CuS-AB/Au/DNA apt)	Electrochemical	DNA	0.1 pM–1 nM	20 fM	[120]
Silver nanoparticles modified graphite-like carbon nitride-based thrombin aptamer (AgNPs-gr/C_3_N_4_ apt)	Electrochemical	Thrombin	100 fM–20 nM	38 fM	[121]
Quaternary CuInZnS quantum dots modified Au nanoparticles-based adenosine aptamer (CulnZnS-QDs/AuNPs apt)	Fluorescence	Adenosine	50–400 µM	1.1 µM	[122]
Silver nanoclusters based complementary DNA aptamer (AgNCs-cDNA apt)	Fluorescence	Lysozyme	2–25 nM	5.6 nM	[123]
Au electrode coated mesoporous silica film/silver nanoparticles based-streptomycin aptamer (MSF/Au/AgNPs strept apt)	Electrochemical	Streptomycin	1 fg/mL–6.2 ng/mL	0.33 fg/mL	[124]

## Data Availability

Not applicable.
